# Ectopic posterior mediastinal thyroid: a case report

**DOI:** 10.1186/1757-1626-1-53

**Published:** 2008-07-21

**Authors:** Sami Karapolat, Ismet Bulut

**Affiliations:** 1 Department of Thoracic Surgery, State Hospital, Bitlis, Turkey; 2 Department of Chest Diseases, State Hospital, Bitlis, Turkey

## Abstract

**Background:**

Posterior mediastinum is a very rare site of ectopic thyroid and such cases are usually identified incidentally by radiography.

**Case presentation:**

A 74 year-old Caucasian male was operated for a mass located in the right posterior mediastinum. Diagnosis of thyroid tissue was confirmed intra-operatively by frozen section and the mass was resected totally. He is well without any problems for 1 year after operation.

**Conclusion:**

Surgical resection may deliver a cure for ectopic posterior mediastinal thyroid and offers good prospects for prognosis.

## Introduction

Ectopic posterior mediastinal thyroid is a rare clinical entity. It comprises some 1% of all mediastinal tumors [1]. Because of silent clinical findings, it is difficult to diagnose clinically. Therefore, they remain asymptomatic for many years, until the mass becomes larger in size. The purpose of this paper was to report this case of ectopic thyroid because of its rarity.

## Case Report

**Figure 1:**
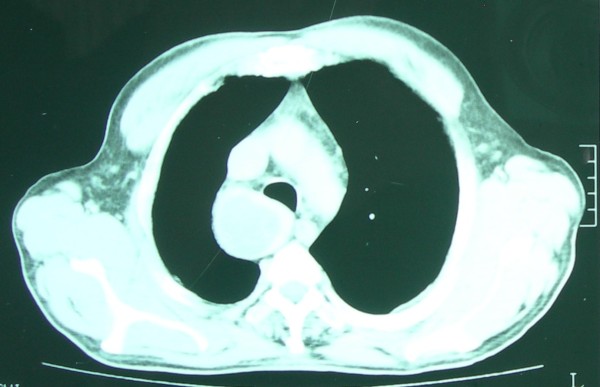
Computed tomographic scan reveals a well-encapsulated mass located at the right posterior mediastinum.

A 74 year-old Caucasian male was admitted to our hospital for medical treatment of severe peripheral arterial disease. A chest pain developed during this time prompted us to conduct laboratory tests. A chest roentgenogram revealed enlargement of the superior mediastinum. In thoracic computed tomography; a homogeneous mass with well-defined borders, measuring 5 × 5 cm, and compressing the trachea was identified in the right posterior mediastinum (Figure 1). The laboratory data including thyroid function tests were within the normal limits. In bronchoscopy, there was no evidence of any endobronchial lesion. Transbronchial fine needle aspiration biopsy was tried but it was unsuccessful.

**Figure 2:**
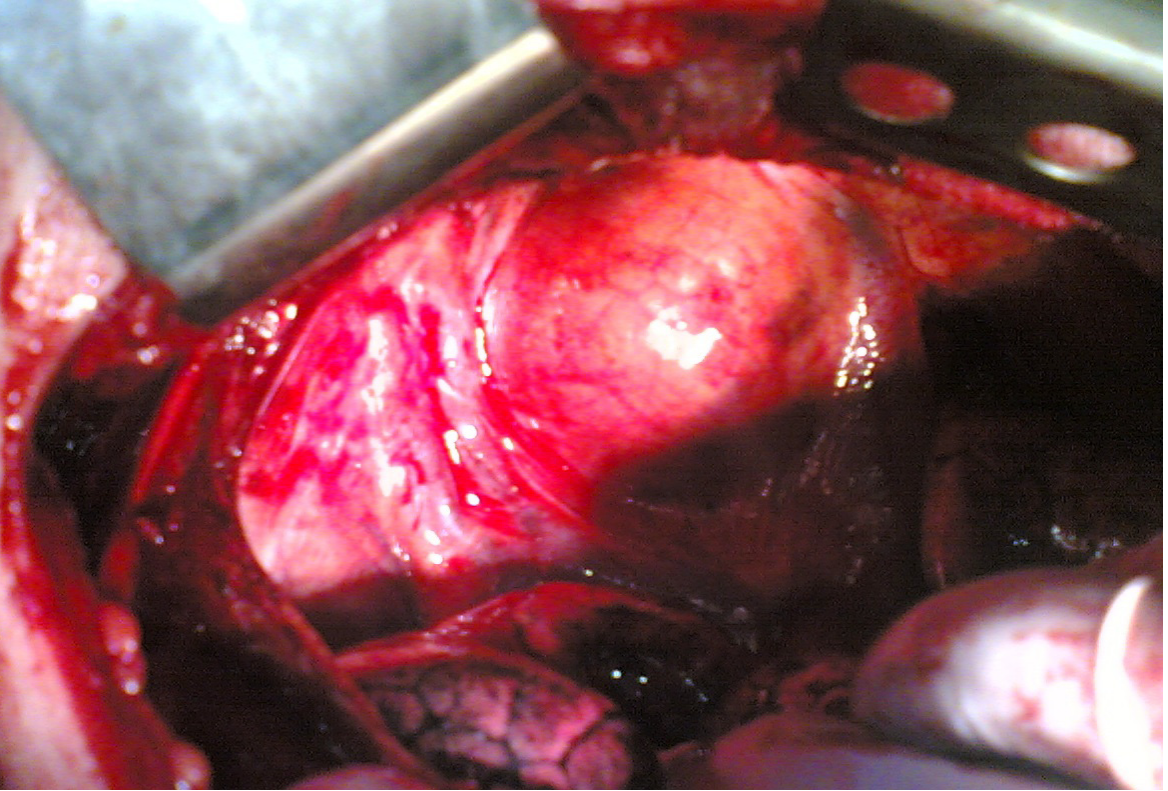
Perioperative view of the mass.

We performed a right thoracotomy and observed a solid mass located in the superior aspect of the right pulmonary hilus (Figure 2). After opening the mediastinal pleura, intrathoracic vessels supplying the mass on its inferior surface was ligated. A biopsy was taken from the mass and sent for frozen section. Histopathological examination revealed thyroid tissue. The mass was resected totally.

**Figure 3:**
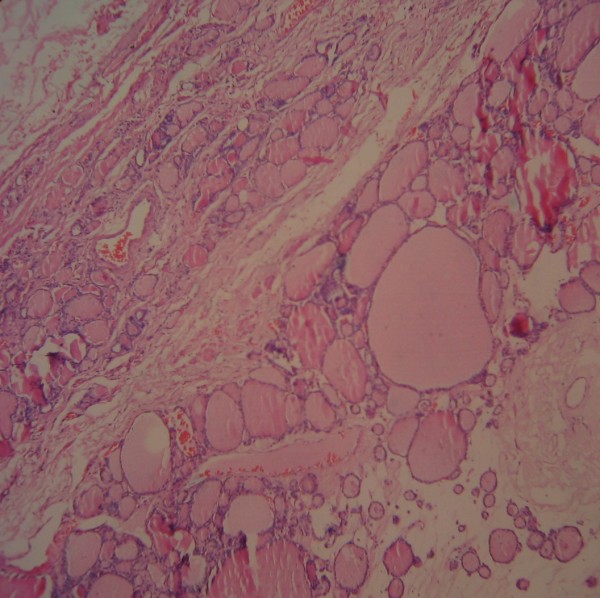
**Histologically, the lesion was diagnosed as thyroid tissue.** (Hematoxylin & Eosin stain, original magnification × 200).

Postoperative pathological examination confirmed previous diagnosis and reported a follicular structure separated into nodules, with cubic epithelium, and whose lumens were filled with colloid (Figure 3). The patient was discharged after recovery on the day 6 of surgery. He was doing well at the 1 year follow-up visit.

## Discussion

Neurogenic tumors, Castleman disease, bronchogenic cysts, Bochdalek's hernia and mesenchymal tumors comprise a great proportion of masses localized in the posterior mediastinum. Though seen rarely, ectopic posterior mediastinal thyroid should also be included in diagnostic possibilities.

It is a benign condition and localized either retrotracheally or retroesophageally. In general, it occurs due to descent of a posterolaterally enlarging inferior pole of the thyroid gland. There may be a displacement in thyroid tissue due to their connection with these during the migration of large vessels in embryogenesis [2].

Ectopic posterior mediastinal thyroid is often asymptomatic. Patients are usually euthyroid. However, symptoms related to the compression on adjacent organs, cough, dyspnea, wheezing, dysphagia, and obstruction of the superior vena cava may be seen. Occasionally, acute tracheal obstruction and severe respiratory failure may be observed [3]. It is usually diagnosed incidentally during radiological procedures performed for other reasons, as in our case.

True malignant transformation in ectopic thyroid tissue is extremely rare [4]. Nevertheless, these masses should be resected surgically due to the risks of malignant transformation, progressive enlargement, hemorrhage within the mass causing respiratory failure, and compression of neighbouring vital mediastinal organs. In the surgical approach, thoracotomy provides both surgical convenience and allows a complete resection with easy access and better visualization. This is a safe procedure with a very low mortality rate and an acceptable morbidity. Finally, complete resection is necessary for achieving a cure.

It usually gets anomalous blood supply from the major great vessels in thorax, especially from the aorta and may show adhesions to surrounding tissues. Therefore, these arterial structures must be ligated and dissection should be performed carefully not to injure the vital organs such as the trachea and the esophagus. In our case, right recurrent laryngeal nerve was adhered to the mass inferiorly and the mass was removed after the nerve was carefully separated from it. Blunt digital dissection without visual control may damage this nerve neighboring the mass and cause vocal cord paralysis postoperatively.

## Conclusion

Although ectopic posterior mediastinal thyroid is a rare entity, it must be considered in the differential diagnosis of posterior mediastinal masses. Surgery is the treatment of choice and prognosis is excellent following complete resection.

## Consent

Written informed consent was obtained from the patient for the publication of this case report and for the use of images. A copy of the written consent is available for the Editor-in-Chief of this journal.

## Competing interests

The authors declare that they have no competing interests.

## Authors' contributions

SK carried out the operation, and prepared the manuscript. IB carried out the preparation of the figures and finalised the manuscript. All authors read and approved the final manuscript.
